# COVID-19: a “black swan” and what animal breeding can learn from it

**DOI:** 10.1093/af/vfaa046

**Published:** 2021-02-05

**Authors:** Henner Simianer, Christian Reimer

**Affiliations:** Animal Breeding Group, Center of Integrated Breeding Research, University of Goettingen, Goettingen, DE

**Keywords:** animal breeding, climate change, COVID-19

**Implications**

Apart from the immediate impact of the COVID-19 pandemic on the breeding industry, there are some lessons to be learned for the future:

- Livestock industry, including animal breeding, has a considerable zoonotic potential and thus must act in a responsible and transparent manner.
- Governments are prepared to take massive action when higher goods are at stake, and the livestock industry will not be exempt from such measures.
- Breeding programs that strive for maximum efficiency are highly vulnerable, so resilience should be included as a strategic goal of breeding operations.
- Global heating is likely to be the next major crisis to develop with major impacts on the livestock sector; livestock breeding should be prepared for it.

In economics theory, the “black swan” metaphor (Taleb 2010) is used for a highly improbable and, thus, virtually unpredictable event, which, when it materializes, has a disruptive impact on the markets. The COVID-19 pandemic can be considered as such a “black swan” event, which not only pushed the global economy in dire straits but primarily had devastating effects on the living conditions on a global scale, including a death toll of currently—October 2020—slightly above a million and many millions more having suffered grave illness. However, was it really so highly improbable and entirely unpredictable that such a pandemic will hit the globe at one stage? When looking at the literature (see, e.g., Jones et al. 2008; Morse et al. 2012; Han et al. 2016), there is a long documented history of zoonoses that turned into a pandemic, with steadily increasing frequency. The U.S. Center for Disease Control and Prevention stated that “... more than 6 out of every 10 known infectious diseases in people can be spread from animals, and 3 out of every 4 new or emerging infectious diseases in people come from animals” (https://www.cdc.gov/onehealth/basics/zoonotic-diseases.html). The intensifying emergence of infectious pathogens is driven by the growing anthropogenic impact on nature and can be attributed, among other factors, to biodiversity loss and habitat degradation (Schmeller et al. 2020), processes which are partly pushed by ever-intensifying livestock production. Many of the risk factors like overpopulation, wildlife markets, and poor hygienic and medical standards are prevalent in developing or emerging countries. Since we live in a highly mobile and globalized world, a local outbreak anywhere in the world, if not immediately detected and consequently eradicated and epidemiological parameters of the causal agent being favorable, has a considerable chance of spreading globally to become a pandemic.

So, all insiders could have been fully aware that a pandemic of major severity might occur at one point of time (just as “the big one” earthquake in the Bay Area will happen at some point) and, at least in theory, plans for such a case were made in many countries. So COVID-19 was not exactly a black swan, although its impact on all aspects of life was very much so, demonstrating that all the emergency plans being developed for such cases, despite being useful to some extent (with a huge variety between countries, though), cannot entirely prevent the adverse effects.

Evidently, the COVID-19 pandemic had a direct impact on the animal breeding industry, with a ban of large meetings making events like animal shows or auctions practically impossible, restriction of mobility of persons and goods (including animals) across borders, but also partly within countries, and forcing people to work from home, which posed an extra hurdle to a direct exchange in teams, etc. Not all affects were exclusively negative, though: by replacing duty travels through video conferences, we all learned that it is possible to organize an exchange with many people from different partners at short notice, which otherwise had to be planned long in advance and with substantial unproductive travel time (and, by the way, a significant carbon footprint). Further effects are indirect in that international trade with animal products partly collapsed, which had a downside effect on livestock production and will have an impact on the demand for breeding products. Compared to other industries (like, e.g., aviation, tourism, or event management), however, the effects on the animal breeding industry so far were relatively mild, meaning that COVID-19 regulations made daily business more complicated and possibly less profitable but, by and large, did not threaten breeding companies or organizations to lose their business model in the short term.

But still, the COVID-19 pandemic can be seen as a warning shot across the bows, thus being a good opportunity to reconsider the situation and the level of preparedness for comparable (or even more severe) crises that we may be faced with in the future. In the following, we will suggest and discuss some hypotheses that might be worth considering when making strategic plans in the animal breeding industry. Admittedly, this is done from a personal perspective with a certain bias toward the German situation.

- *In a pandemic situation, the livestock industry as a whole, and animal breeding as part of it, may be seen as very critical by the general public.*
  The majority of severe pandemics are caused by close contact between humans and animals, and the proportion of zoonoses transmitted by farm animals rather than wild animals has steadily increased over the years (see Jones et al. 2008). Therefore, animal breeding as part of animal production will be considered particularly skeptical by the general public as a potential source of zoonosis-associated pandemics. In the worst case of a pandemic caused by a zoonosis transmitted by farm animals, comprehensive documentation and traceability of animals and products is of key importance. Overall, livestock production for diverse reasons has a poor reputation in many Western societies, and events like the current pandemic have the potential to pull production conditions even more into the focus. In the COVID-19 pandemic, slaughterhouses and meat packaging plants proved to be super-spreading platforms, provoking a very critical review of the sometimes actually reprehensible working conditions there. Such circumstances can foster the already existing alienation between the general society and animal production, with long-term consequences, for example, for production standards and market demand for animal products in competition with plant-based diets or meat substitutes. The livestock industry, including the breeding sector, should always be aware that it is under critical and sometimes hostile observation, and anything that potentially deepens this alienation should be avoided.
- “A ‘black swan’ event can have major disruptive effects on various crucial activities of animal breeding programs.”
  We now have with the COVID-19 pandemic, a specific case with specific consequences for daily life and business as described above. We have learned that effects can be much more extreme than expected: who would have thought that it is possible to implement—from one day to another—a complete halt of international travel or to put whole countries effectively into a “stay at home” quarantine for weeks or months? We should be aware, though, that another “black swan” may have very different consequences, possibly even more severe or affecting other aspects of life and business. Just to give an example from a rather different realm: shortly before Christmas last year, a major German university was attacked with the malicious computer virus “Emotet.” Despite having a professional cyber security infrastructure in place, the entire computer system had to be shut down—essentially by pulling the plug—and it literally took weeks before at least some of the systems could be gradually restarted. Note that this not only affected scientific work but the entire organization, including personnel administration, student admission, procurement, and bookkeeping, no emails could be sent and received etc. You do not need much fantasy to imagine the devastating impact such a scenario would have on a modern large-scale breeding operation.
- “While it is not possible to be perfectly prepared for all eventualities, there are ways to improve and verify the level of preparedness.”
  We are sure all breeding companies have their emergency plans in place, but we should be aware that it will not be possible to be fully prepared for all eventualities. It might be a good opportunity, though, to review these plans now and possibly think of even more extreme scenarios based on the current experience. In the financial world, regular “stress tests” are undertaken, in which a hypothetical scenario mimicking a financial crisis is simulated in a very realistic and detailed way and then banks are assessed as to whether they are sufficiently prepared so that they can handle, and actually ultimately survive, such a scenario (see, e.g., Upper 2011). It might be a good idea to consider a similar “stress test” approach for breeding companies and see how they would do.
- “Operations striving for maximum efficiency run the risk of high vulnerability, so we should include resilience as a strategic goal of breeding operations.”
  As in many other fields of business, breeding programs over the last decades have been continuously pushed toward maximum efficiency. It is taught at universities, including ours, that maximizing genetic progress or better, maximizing breeding efficiency—basically genetic progress per unit of capital input—is the primary objective. High efficiency inevitably comes along with high levels of complexity: think of the complex infrastructure in performance testing and data management but also the complex logistics when it comes to transferring the genetic progress made in the breeding nucleus to the practical farms. In the context of the COVID-19 pandemic, it became evident that high efficiency comes at the expense of resilience, which makes the most efficient, and in “normal times” most successful, enterprises most vulnerable and, thus, more at risk, when shocks occur (see, e.g., the interesting opinion piece of W. J. Galveston in the Wall Street Journal; https://www.wsj.com/articles/efficiency-isnt-the-only-economic-virtue-11583873155). So, there is an obvious trade-off between efficiency and resilience. But are we not, as animal breeders, experienced in operating with trade-offs between conflicting goals, such as performance and fitness, in breeding? Consequently, we should apply the same basic principles in the design of resilient breeding programs and operations.

**Figure:**
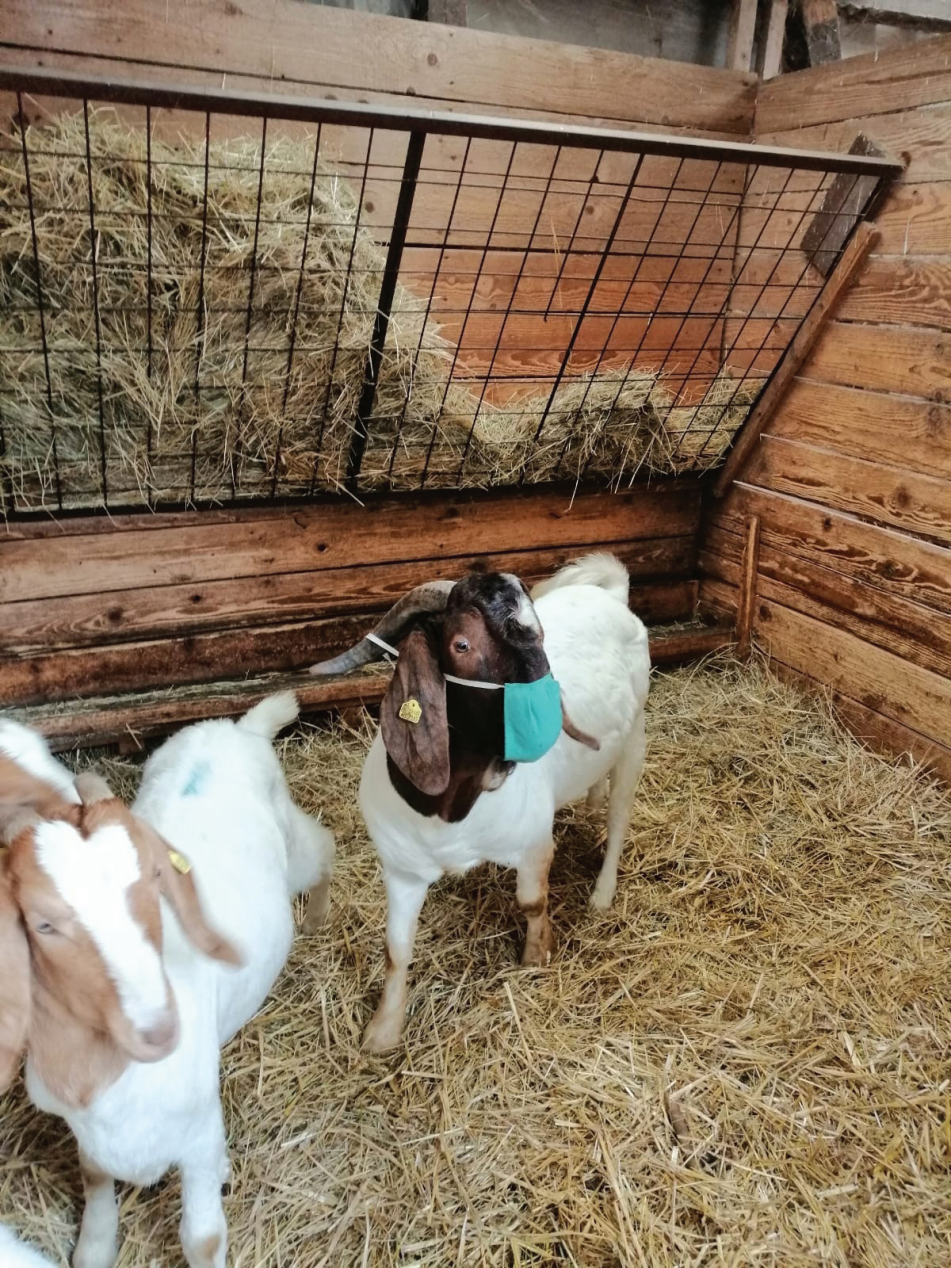
Goat with COVID mask.

The COVID-19 pandemic often is compared with the “Spanish flu” pandemic almost exactly a century ago and, thus, one might hope that it is a “once in a century” event and we might be safe for another century when we have overcome the current crisis. It is needless to say that this view is naïve and unrealistic. Actually, we even know that another “black swan” is steadily approaching, which is global heating (which in our view is the more appropriate term compared to the neutral “climate change”). Global heating itself and the commitment of 189 countries in the 2016 Paris Agreement to limit the CO_2_ emissions such that the heating will not exceed 2 °C compared to the preindustrial level will have an impact on the livestock industry that hardly can be overstated (Rojas-Downing et al. 2017). Livestock production will not only be affected in places where environmental changes, like droughts, have a direct impact, but there will be an overall change in the way how businesses can operate everywhere in the world. Each country has a certain emission budget, and it will have to make a decision how to spend it wisely. As we have learned in the current pandemic, governments are prepared to act massively when higher goods are at stakes. The Netherlands, for instance, have decided in 2019 to introduce a 100 km/h speed limit on motor highways and to compensate pig producers to give up their business in order to be able to continue other emission-intensive activities like building houses. Ultimately all products and production processes will be assessed and taxed according to their carbon footprints along the entire value chain. This is going to happen within the next few years or decades, and it likely will have a major effect at least on some segments of the livestock industry. Again, as in the COVID-19 pandemic, this will not be an entirely improbable and, thus, virtually unpredictable event (so, no classical “black swan”), but it still will have disruptive effects in many areas. Livestock breeding should be prepared.

*Conflict of interest statement.* None declared.

## About the Authors

**Henner Simianer** is Professor of Animal Breeding and Genetics at the University of Goettingen, Germany. Coming from a quantitative genetics background his interest lies in the dynamics of the genetics underlying complex traits. He further has made major contributions to the optimum design of breeding programs. Recently, he widened his interest to reveal methodological synergies between animal and plant breeding, which led to the establishment of the Center of Integrated Breeding Research (CiBreed) at the University of Goettingen.

**Christian Reimer** originates from a rural area in Northern Germany and studied agricultural sciences at the University of Goettingen with a focus on animal sciences. He then joined the Animal Breeding and Genetics group as a PhD student and graduated with a thesis entitled “Sequence-based Analyses of the Goettingen Minipig Genome”. As a post-doc, he has a research focus on genomic evaluations as well as the optimization of breeding programs with complex breeding goals.

## References

[CIT0001] Han, B. A., A. M. Kramer, and J. M. Drake 2016 Global patterns of zoonotic disease in mammals. Trends Parasitol. 32:565–577. doi:10.1016/j.pt.2016.04.007 27316904 PMC4921293

[CIT0002] Jones, K. E., N. G. Patel, M. A. Levy, A. Storeygard, D. Balk, J. L. Gittleman, and P. Daszak 2008 Global trends in emerging infectious diseases. Nature 451:990–993. doi:10.1038/nature06536 18288193 PMC5960580

[CIT0003] Morse, S. S., J. A. Mazet, M. Woolhouse, C. R. Parrish, D. Carroll, W. B. Karesh, C. Zambrana-Torrelio, W. I. Lipkin, and P. Daszak 2012 Prediction and prevention of the next pandemic zoonosis. Lancet 380:1956–1965. doi:10.1016/S0140-6736(12)61684-5 23200504 PMC3712877

[CIT0004] Rojas-Downing, M. M., A. P. Nejadhashemi, T. Harrigan, and S. A. Woznicki 2017 Climate change and livestock: impacts, adaptation, and mitigation. Clim. Risk Manage. 16:145–163. doi:10.1016/j.crm.2017.02.001

[CIT0005] Schmeller, D. S., F. Courchamp, and G. Killeen 2020 Biodiversity loss, emerging pathogens and human health risks. Biodiversity and Conservation 29(11–12):3095–3102. doi:10.1007/s10531-020-02021-6 PMC7423499 32836920

[CIT0006] Taleb, N. N 2010 The Black Swan: the impact of the highly improbable. Rev. ed. London, UK: Penguin Books.

[CIT0007] Upper, C 2011 Simulation methods to assess the danger of contagion in interbank markets. J. Financial Stability 7(3):111–125. doi:10.1016/j.jfs.2010.12.001

